# COVID-19 Vaccine Collaborative Supply Planning: Is This the Next Frontier for Routine Immunization Supply Chains?

**DOI:** 10.9745/GHSP-D-23-00150

**Published:** 2024-02-20

**Authors:** Laila Akhlaghi, Wendy Prosser, Avotiana Rakotomanga, Janet Makena, Tochukwu Azubike, Yahaya Bello, Samson Emelike, Liteboho Mothetsi, Moroke Motuba, Victor Olayemi, Sam Samba, Silvestre Suh, Stevens Ramaroson, Fatimata dit Ngo Yarro

**Affiliations:** a John Snow, Inc., Arlington, VA, USA.; b John Snow, Inc., Antananarivo, Madagascar.; c inSupply Health, Nairobi, Kenya.; d Ministry of Health, Abuja, Nigeria.; e John Snow, Inc., Abuja, Nigeria.; f John Snow, Inc., Maseru, Lesotho.; g Clinton Health Access Initiative, Maseru, Lesotho.; h Clinton Health Access Initiative, Freetown, Sierra Leone.; i John Snow, Inc., Bamako, Mali.

## Abstract

In response to the COVID-19 pandemic, vaccine manufacturers quickly pivoted to develop vaccines, offering limited visibility into production timelines and quantities. Low- and middle-income countries developed vaccine introduction plans within the context of constrained global supply, unknown vaccine availability or timelines, and unknown demand. The Vaccine Collaborative Supply Planning (VCSP) Initiative was established to address the gaps in visibility of supply and demand of COVID-19 vaccines for improved supply planning. The Initiative drew from experiences with supply planning for other health program products. The Initiative’s 2 goals for introducing COVID-19 vaccines were to: (1) incorporate the use of supply chain data for forecasting, supply planning, and resupply decisions; and (2) establish a collaborative model for governments and partners to learn and implement. Beginning in September 2021, the Initiative was operational in 15 countries through strategic partnerships with government immunization programs and 7 partnering organizations. Partners noted the Initiative offered several benefits: enabled visibility into stock status; provided ability to triangulate service delivery and stock data; provided insight into products at risk of expiry; facilitated tracking of monthly consumption patterns to inform decisions; offered ability to create forecasting scenarios; provided support to national logistics working groups; improved coordination at the country level; created trust through transparency across global partners; and offered opportunity for real-time adjustments through adaptive learning. Partners also noted challenges, including the influence of political decisions and the availability of quality data. The Initiative successfully applied best practices for supply planning, established a collaborative model with government decision-makers and partners, and used an adaptive learning approach to respond to the challenges of rolling out the COVID-19 vaccine. Lessons learned and recommendations suggest how this approach can be used to strengthen forecasting and supply planning for all vaccines as the COVID-19 vaccine is integrated into routine services.

## Abstract (precis)

Collaborative supply planning approaches established for the COVID-19 vaccine roll-out can be adapted to routine immunization for improved vaccine management.

## Teaser

**Key Findings:**

- To support the COVID-19 vaccine roll-out, a collaborative partnership, the Vaccine Collaborative Supply Planning Initiative, developed supply planning approaches building upon experiences from other health program products to review past consumption patterns and apply them to understanding inventory situations, requirements, and shipments.
- This Initiative applied best practices for supply planning, established a collaborative model with government decision-makers and partners, and used an adaptive learning approach to respond to the challenges of rolling out the COVID-19 vaccine.

**Key Implication:**

- Immunization program managers can adapt this supply planning approach for routine vaccines to improve visibility into consumption and stock status and contribute to strengthening the overall management of the immunization supply chain.

Plain language article summary available.

## BACKGROUND

The COVID-19 pandemic’s disruptive effects in early 2020 prompted a global effort to both reduce the immediate spread of the virus and develop effective vaccines to end the pandemic quickly. By mid-2021, preclinical and clinical development of almost 300 COVID-19 vaccine candidates was underway, with regulatory approval for emergency use given to 6 vaccines, which were then deployed worldwide.^1^ However, despite significant scaling efforts, limited manufacturing production capacity and input shortages—compounded by disruption in global supply chains—resulted in an opaque landscape for production timelines and quantities. Additionally, countries producing the vaccine prioritized domestic use, initially restricting exports to other countries, leaving many low- and middle-income countries (LMICs) without access to the vaccines.^2^

The global community took several measures to address the uneven supply and distribution of COVID-19 vaccines. The Vaccine Trade Tracker was established to monitor production across all manufacturers,^3^ and dose-sharing commitments were made to increase the flow of vaccines to LMICs.^4^ Gavi, the Vaccine Alliance, created the COVAX Facility to accelerate rapid vaccine production and ensure equitable access for all countries.^5^ Within the facility, the Gavi COVAX Advance Market Commitment is a separate funding mechanism, particularly for LMICs that heavily relied on donations to obtain vaccines. COVAX initially prioritized high-risk, vulnerable populations and essential health workers due to supply constraints. They designed the allocation plan for vaccine distribution to LMICs based on population size.

Country COVID-19 coordination teams in LMICs, typically led by the Essential Programme on Immunization (EPI) within the ministries of health (MOHs), prepared for the new vaccine by developing national vaccine deployment plans. These plans defined distribution and campaign strategies, identified priority populations, and set targets for coverage.^6^ Vaccination plans were mostly designed as large-scale campaign efforts aimed to reach hundreds or even thousands of people daily in central locations with large vaccination teams with all the required supplies. However, the national vaccine deployment plans were developed in the context of constrained supply, unknown vaccine availability or timelines, and unknown demand for these vaccines. The unknown demand was because the target population was different from populations that traditionally access immunization programs for routine immunization, which complicated the ability to ensure the availability of adequate stock levels. Furthermore, LMICs could have received vaccines through COVAX or other donors, bilateral donations directly from manufacturing countries, and some government self-procuring of vaccines, complicating the global planning perspective.

In early 2021, the global community realized that it required new coordinating and planning approaches to ensure equitable vaccine availability where needed.

In early 2021, the global community realized that it required new coordinating and planning approaches to ensure equitable vaccine availability where needed.

The Vaccine Collaborative Supply Planning (VCSP) Initiative was established to address the gaps in visibility of both the supply and demand side of COVID-19 vaccines for improved forecasting and supply planning. Forecasting is the process of estimating the quantities of products that will be dispensed or used. Supply planning involves determining the total quantities of products required, taking into account forecasted consumption, existing product in the pipeline, desired stock levels, shipping lead times, and desired arrival dates of shipments.^7^ We document the rationale and global best practices that drove the design of the VCSP Initiative; describe the tools and collaborative approach; and provide evidence of success, feasibility, and acceptability, and potentially its use as a foundational approach for all vaccines, as immunization programs are shifting to integrate COVID-19 into routine services.

## VCSP INITIATIVE DESIGN AND DESCRIPTION

Noting the need for more robust supply planning to be used in practice with the evolving immunization decisions, John Snow, Inc. (JSI), as the lead partner working with MOHs, EPI, and key partnering organizations, drew from its experiences with supply planning for other health program products in designing the VCSP Initiative, particularly when faced with supply constraints and the need for collaboration. The Initiative had 2 primary goals for introducing COVID-19 vaccines in participating LMICs (Table 1): (1) to incorporate the use of supply chain data for forecasting, supply planning, and resupply decisions; and (2) to establish a collaborative model for governments and partners to learn and implement. Achieving these goals would make the case for these approaches for routine immunization and integration of the COVID-19 vaccine services into primary health care.

**TABLE 1.** Vaccine Collaborative Supply Planning Initiative Participating Countries and Partner Organizations

**Participating Country**	**Partner Organization**
Democratic Republic of Congo	VillageReach
Côte d’Ivoire	CHAI
Ethiopia	JSI
Kenya	inSupply Health Limited
Lesotho	CHAI
Madagascar	JSI
Malawi	JSI
Mali	JSI
Mozambique	VillageReach
Niger	JSI
Nigeria	JSI
Sierra Leone	CHAI
South Africa	Guidehouse
Tanzania	inSupply Health Limited
Uganda	PATH

Abbreviations: CHAI, Clinton Health Access Initiative; JSI, John Snow, Inc.

### Incorporate the Use of Supply Chain Data to Drive Forecasting, Supply Planning, and Resupply Decisions

During the design phase, the VCSP Initiative identified several technical priorities for forecasting, supply planning, and resupply decisions at both the country and global levels. These priorities were informed by the Global Family Planning Visibility and Analytics Network (GFPVAN, https://www.rhsupplies.org/gfpvan/), which was created in 2012 to improve coordination for the supply of family planning products. The GFPVAN established a collaborative space to track and fine-tune the flow of products across multiple countries and coordinate with multiple donors (i.e., to avoid last-minute requests from countries, ensure product availability at the country level, minimize overstock and expiries, and provide more stable predictability of the product need for manufacturers), resulting in improved product availability, proactive supply planning and budgeting by governments, and cost savings of more than US$100 million.^8^

Based on the experience of the GFPVAN and tracking the challenges with the introduction of the COVID-19 vaccine, the Initiative prioritized country-led decisions and insights, ensuring data visibility to support those decisions. The VCSP Initiative aimed to shift the decision-making powers to country leaders having and using their own data and evidence to forecast and plan their vaccine needs based on actual demand, experience with mass campaigns, and a clear understanding of the stock status in their countries.

The VCSP Initiative aimed to shift decision-making power to country leaders using their own data and evidence to forecast and plan their vaccine needs based on actual demand, experience with mass campaigns, and a clear understanding of the stock status in their countries.

The VCSP Initiative achieved this by developing a country-based decision tool that adjusted the data collection and analysis typically done in an immunization program to be more appropriate for the dynamic supply and demand experience of COVID-19 vaccines. The tool was designed, validated with country-level stakeholders, and continuously revised based on feedback to improve usability and analytics for decision-making. The country teams were initially trained on the tool, and regular check-ins reinforced its use and provided an opportunity to clarify any questions and/or adapt the tool. The tool reports and uses monthly consumption, which reflects a feasible expectation for the number of vaccines administered and people reached through campaign (or noncampaign) efforts. With this insight, decision-makers can review historical patterns to estimate how long the current stock will last (with potential expiry estimates) and when and how much new stock is required. They can also use this information to determine different scenarios for campaign efforts and better manage and prioritize stock rotations based on potential expiries. This analysis provides the ability to change and update orders and supply plans to ensure a steady and predictable supply.^9^

The Initiative also prioritized creating a standardized, open, and transparent process across public, private, and government stakeholders. The GFPVAN put a considerable amount of effort into creating a network of partners, redefining processes for forecasting and supply planning, and identifying governance structures that were beneficial for all stakeholders. With that in mind, the VCSP Initiative created a similar standardized process and naming convention, aligned stakeholders on data-sharing expectations, and collectively developed a data-sharing agreement. Data were anonymized for noncountry stakeholders, and reporting and sharing mechanisms for global partners were established for insights that should be considered for global planning.

As the largest procurers of vaccines in LMICs, COVAX closely tracked country-level consumption and potential expiries, constraints, and stock-outs of vaccines they procured. However, COVAX could not account for bilateral arrangements directly between countries, direct donations, or other sources such as the African Vaccine Acquisition Trust. The VCSP Initiative approach prioritized a system that reports on and tracks all COVID-19 vaccines procured by or donated to countries; the aggregate data provides greater global visibility than any single donor could without collaboration. The Initiative’s approach provides country-level insight to strengthen global planning, which, in turn, benefits countries.^10^

### Establish a Collaborative Model for Governments and Partners to Learn and Implement

The global effort to get COVID-19 vaccines to billions of people around the world required a collaborative approach among multiple stakeholders. Such partnerships are most effective when partners who are aligned on shared goals and purpose build understanding and trust and use common planning processes while investing in connections.^11^^–^^14^

From the beginning, the VCSP Initiative was designed as a collaborative model that leverages the strengths, expertise, and connections of all partners, with the government immunization program in the lead. JSI worked with partners to introduce the project and approach, and then countries self-selected for participation based on government and in-country partner interest. The Initiative identified strategic partners in priority countries that had strong relationships and demonstrated trust with the MOH and the EPI supply chain team. The team, which included Clinton Health Access Initiative, Guidehouse’s Global Health Supply Chain-Technical Assistance project, inSupply Health, PATH, VillageReach, and led by JSI, worked closely with the National Logistics Working Group (NLWG) in each country as the coordinator for the vaccine supply chain. EPI largely used mass campaigns to reach the most people in an efficient way with the new vaccine. Partners had supply chain expertise but not necessarily extensive experience with supply planning. The VCSP Initiative also prioritized building and maintaining relationships with global partners, such as Gavi, COVAX, the World Health Organization, UNICEF, and the U.S. Agency for International Development. Within 8 months of starting, the VCSP Initiative organized a retreat to build a community of vested and trusted partners aligned on a common goal and purpose for the Initiative, being transparent about challenges faced by countries. A second retreat then continued the objectives to build community by increasing participation, sharing lessons learned, and strategizing on what would be needed for the future of the collaboration.^15^

The VCSP Initiative established regular touchpoints with and between all partners through a WhatsApp group and regular check-ins, creating a network where partners shared information and sought support from other partners directly. The Initiative designed an adaptive learning approach to facilitate continuous learning and optimize program effectiveness.^16^ The approach systematically collected information on the implementation process of the Initiative, collaboration aspects, and the technical aspects of supply planning and vaccine introduction. The VCSP Initiative shared this information with key stakeholders at the appropriate time to ensure understanding and consideration by decision-makers and adjust the approach and tools in real time.

Collectively, the project team across all countries, together with government stakeholders, continuously tracked progress and documented insights into lessons learned and key success factors of the decision tool and collaborative approach. We present the results of demonstrated benefits as well as challenges identified since the start of this Initiative.

## IMPLEMENTATION RESULTS

Since its inception in September 2021, the VCSP Initiative has expanded its operations from 5 to 15 countries through strategic partnerships. Six partnering organizations work closely with the EPI and other in-country partners. The decision tool used the same consumption and supply data available to in-country teams managing the COVID-19 efforts but analyzed it differently, which was more appropriate for a dynamic environment. Partners noted that the VCSP Initiative introduced several innovative ways of data management and analysis that were not previously routinely used in immunization programs, resulting in demonstrable benefits and making the case for integrating these innovative methods of forecasting and supply planning into routine immunization efforts.^17^ Although partners noted the benefits, full adoption of the Initiative into routine immunization efforts requires longer-term effort, policy changes, ongoing technical support and capacity-building, and global guidance beyond the scope of this project. The VCSP team also noted ongoing challenges that the Initiative faced during implementation. It is important to note that the indicators and visuals presented are only simulated data and are not reflective of any country. They are included as an example of how this approach is applied and how the data is used.

Partners noted that the VCSP Initiative introduced innovative ways of data management and analysis, resulting in demonstrable benefits and making the case for integrating these innovative methods of forecasting and supply planning into routine immunization efforts.

### Benefits

#### Enabled Visibility Into Stock Status and Months of Stock

**FIGURE 1:**
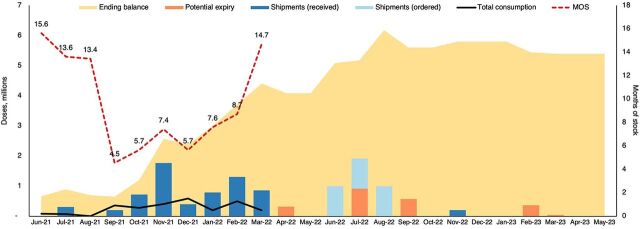
Vaccine Collaborative Supply Planning Tool Dashboard Chart Displaying the Summary of Vaccine Stock Status Abbreviation: MOS, months of stock.

Partners noted that the VCSP tool enabled the EPI supply chain managers to determine resupply or order quantities, monitor supply plans and supply chain status, and monitor performance at subnational levels. This allowed them to ensure that there was enough (but not too much) product available to meet the need wherever and whenever it was needed. It did this by providing visibility into the stock status as a relative measurement of the period of time that the stock would last based on average rates of most recent consumption. Decision-makers were also able to determine whether to postpone orders for the sake of avoiding constraints on the cold chain. Additionally, using stock levels and months of stock as a metric allowed for more efficient and effective monitoring of the supply (Figure 1).

#### Provided Ability to Triangulate Service Delivery and Supply Chain Data

**FIGURE 2:**
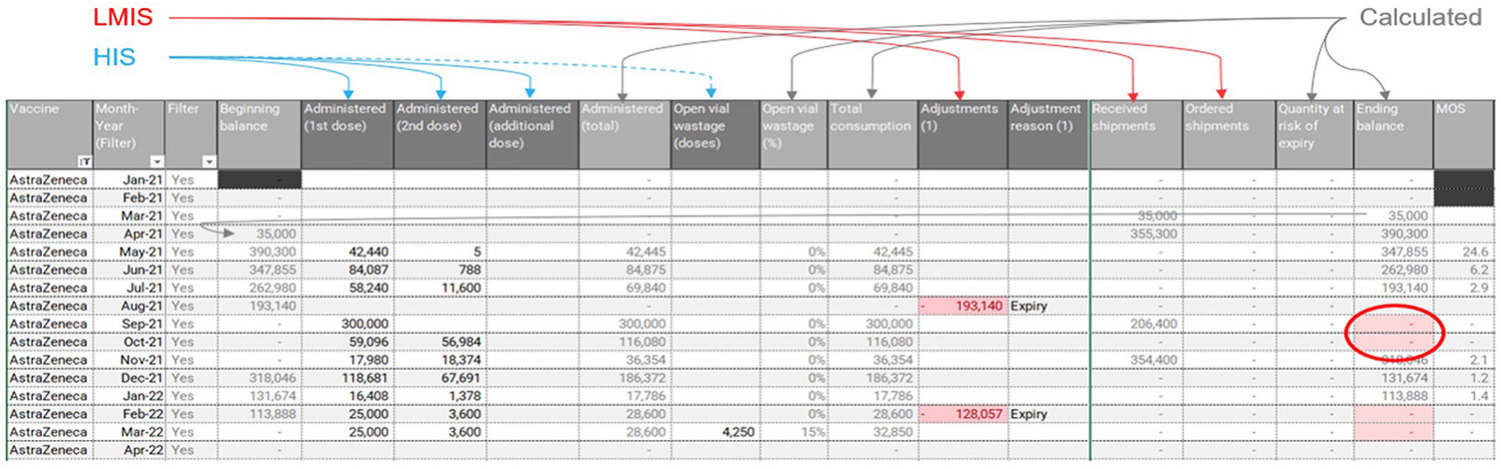
Data Entry Example in Vaccine Collaborative Supply Planning Tool Combining Both Service and Supply Chain Data and Flagging Possible Errors Abbreviations: HIS, health information system; LMIS, logistics management information system; MOS, months of stock.

The VCSP tool combined data from both service delivery and supply chain to provide visualizations that supported decision-making, making it a unique aspect of the tool. Service delivery data, which included vaccine consumption that is calculated using doses administered and wasted, was combined with supply chain data, capturing all incoming shipments with their relevant specifications (e.g., expiration dates) and any adjustments made to the stock level (Figure 2). The decision tool documented any changes to the balance, highlighting data quality issues. For example, the tool could alert users that there was no usable product available to be reported as used (circled in Figure 2). Partners reported that this feature led to careful reviews of both sets of input data and corrective action to improve data quality not just for COVID-19 vaccines but for all vaccines.

#### Gained Insight Into Products at Risk of Expiry

**FIGURE 3:**
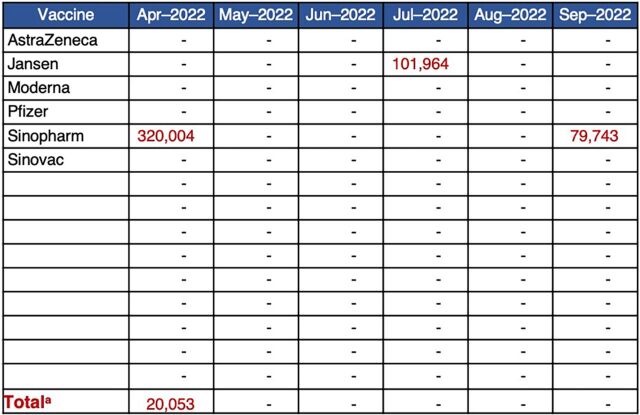
Example of Vaccine Collaborative Supply Planning Tool Dashboard Table Displaying Quantities at Risk of Expiry Given Current Average Consumption Rates ^a^ Total assumes no preference for products being administered.

The process of entering data into the VCSP tool required the inclusion of shipment details, such as expiry dates. The triangulation of data on consumption, stock levels, and estimated expiries by applying the assumption of first-expiry, first-out rules identified when and how many doses had the potential to expire (Figure 3). The analysis of these data allowed countries to prioritize certain vaccines for distribution and campaigns and/or reallocate vaccines at risk of expiry to other countries that could more readily use those vaccines at that moment. Furthermore, to address the short shelf life of vaccines received in the early phases of the vaccine roll-out, countries took measures, such as setting policies for minimum acceptable shelf life from donors and manufacturers.

#### Facilitated Tracking of Consumption Patterns by Month to Inform Decisions Related to Both Supply and Demand

**FIGURE 4:**
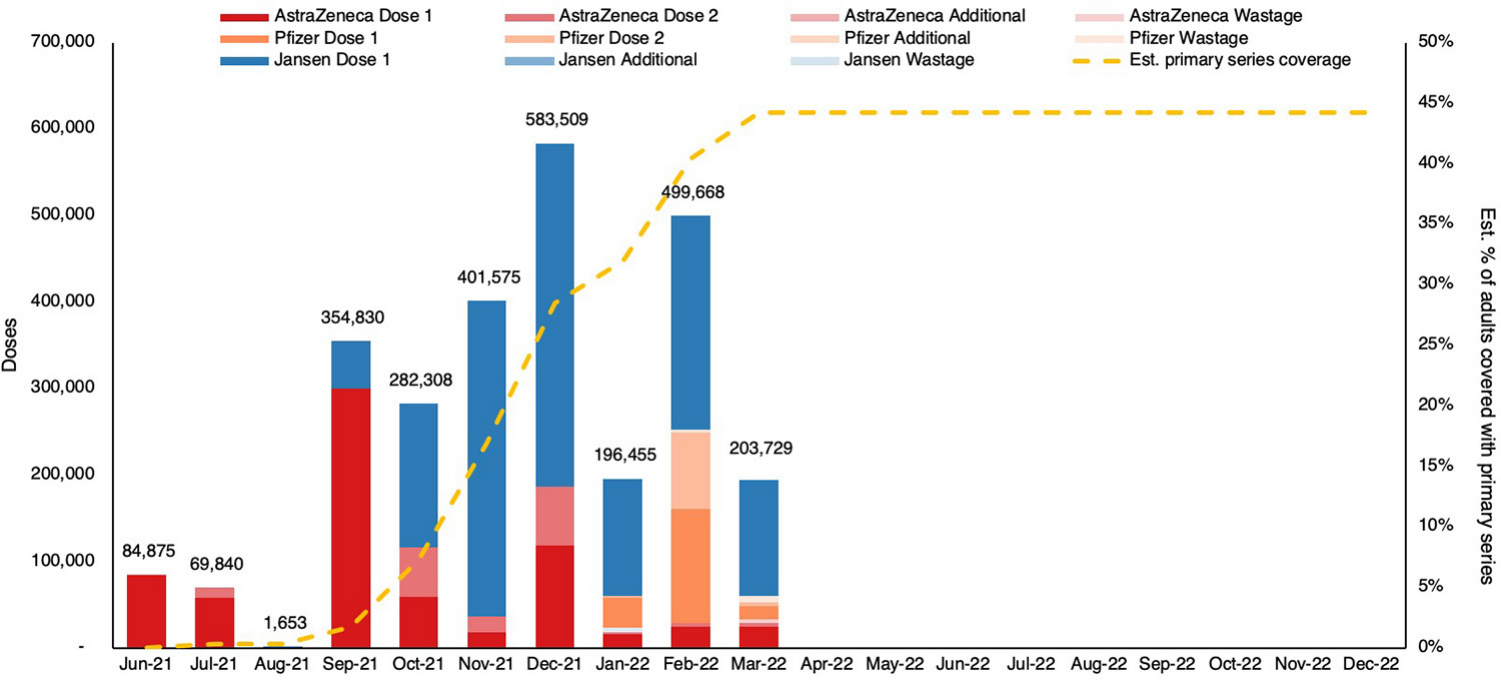
Example of Vaccine Collaborative Supply Planning Tool Dashboard Chart Showing Consumption (Doses Administered and Wasted) of Vaccines Per Month With Coverage of Target Population

Other health programs routinely track monthly consumption, but this type of analysis was not standard practice in immunization programs, as monitoring tended to track coverage rates over time based on aggregate vaccine administration. However, due to the unstable context of the COVID-19 vaccine and the need to adapt processes for the changing cohort receiving the vaccine, there was a demand for additional data to support decision-making. The VCSP decision tool addressed this need by providing monthly consumption patterns that included both doses administered and wasted, allowing for the identification of the effects of campaign efforts on consumption, which aided in understanding future demand, given available resources (Figure 4). This led to more effective demand creation, optimized vaccine distribution to subnational levels, and adjustments to required resources. Partners noted that the tool provided the ability to adjust supply planning in response to emerging issues with better timing of deliveries and preferred vaccines.

#### Offered Ability to Create Various Forecasting Scenarios

Decision tool users could create various scenarios based on consumption patterns, target goals, and realistic expectations of what was feasible to see the effect on stock status and expirations. These scenarios could be used in discussions to proactively determine the quantity and timing of future orders, reject orders, review the magnitude and timing of potential expiries, review changes to coverage rates, and assess the capacity of the system to provide the necessary vaccinations required per month to reach goals. Using the possible outcomes generated by the scenario generation feature, managers could preempt and mitigate potential supply chain risks for all possible outcomes.

#### Provided Support and Reinforcement for NLWG

Partners highlighted that the VCSP Initiative reinforced the role and skills of the NLWG. The introduction of the COVID-19 vaccine required the NLWG to adjust its management style to respond to the complex environment that lacked both visibility into vaccine supply and/or demand from the new cohort of patients. The Initiative provided a structured approach for supply planning, which the NLWGs in the 15 countries adopted. In some instances, the Initiative collaborated with in-country partners to revise the terms of reference of the NLWG, clarifying roles and responsibilities for reviewing stock status. The VCSP Initiative introduced a draft monthly report as part of the standard processes of the NLWG, enabling the NLWGs to elevate supply chain decisions to higher-level decision-makers with the evidence necessary for optimal supply planning. One country also hired new staff for data management for the supply chain based on the new understanding of how the vaccine roll-out was progressing.

#### Improved Coordination at Country Level

Government leaders and immunization program managers engaged in-country partners to support the impressive introduction of COVID-19 vaccines. One benefit that the VCSP Initiative partners noted was the coordination effort required by the VCSP decision tool, which drew data from service delivery as well as supply chain actors and systems. This collaboration required different stakeholders to work together to input and analyze the data. The partners noted that this approach was helpful to other areas of vaccine introduction, including identifying where demand-generation activities would be beneficial and how to best distribute the preferred vaccine based on previous consumption and availability. Through this structured data review and collaborative process, trust was built across partners, contributing to more transparency, increased sharing of data, and, ultimately, more proactive supply planning not only for COVID-19 vaccines but for all vaccines.

#### Created Trust Through Transparency Across Global Partners

The VCSP Initiative made significant efforts to engage all partners in an open and transparent manner at the global level, providing high-level insight into global consumption and supply trends using aggregate country-level data. Country-specific data was shared only by the EPI through standard channels for donor reporting and procurement decisions and was not reported by the Initiative, which contributed to country ownership and reinforced trust in the process and data use.

#### Offered Opportunity for Dynamic Approaches Through Adaptive Learning

The VCSP Initiative developed a self-assessment framework that tracked and measured the evolution of supply planning activities. This was developed through adaptation of the Supply Chain Evolution Framework.^18^ The country teams, which included the VCSP country lead, EPI, and partners, regularly evaluated the country’s progress in COVID-19 vaccine supply planning, moving along the continuum of ad hoc to reactive to proactive response. Technical aspects of supply planning and management were assessed using aspects listed in Table 2. The self-assessment was subjective and allowed teams to critically reflect and analyze their supply planning process to identify the areas that needed improvement to adapt to changing circumstances. Country teams shared their insights with all partners, creating opportunities for cross-country collaboration and learning. Over the course of the Initiative, countries made progress along the evolution continuum toward more proactive supply planning by addressing and prioritizing the weakest technical areas. The assessment results were not used to rank countries based on their performance and were anonymized while sharing them among teams.

**TABLE 2.** Supply Planning and Management Technical Aspects Regularly Reviewed for Adaptive Learning for the Vaccine Collaborative Supply Planning Initiative

**Technical Aspects**	**Characteristics to Rank** **(Ad Hoc–Reactive–Proactive)**
Adjustments	Communicated Funded Implemented
Analysis	Routine assessment Scenario monitoring Supply plans Updated forecasts Vaccine expiry
Data	Access Accuracy Reliable system Reporting practices
Commitment	Resources allocated Stakeholder commitment Stakeholder inclusion Terms of reference existence
Meetings	Actions reviewed Evidence-based decisions Flexibility ad hoc meetings Risks addressed Scheduled Stakeholder inclusion Timely decision-making

Although all 9 benefits are notable, building capacity and providing support for the NLWG may contribute the most to stronger management of the immunization supply chain for all vaccines. This approach introduced new supply planning techniques to the NLWG, bringing in best practices from other health areas not normally considered by EPI and providing an opportunity for growth and skills development.

### Challenges Faced by VCSP

#### Political Decisions Not Informed by Technical Insight

Country COVID coordination teams faced political decisions, such as accepting bilateral donations, which may not have aligned with sound stock management, cold chain capacity, feasibility of large-scale campaigns, or the preference or efficacy of the vaccine. Furthermore, countries’ initial targets for COVID-19 vaccine coverage were established based on several unknowns, including unclear estimates of a new vaccination cohort not previously targeted for vaccines. In addition, targets were developed with the assumption that the country had the ability and the capacity to implement the vaccination plan without commensurate support and resources. Although the VCSP tool could provide evidence of the impact of these politically driven decisions and offer scenarios to minimize risks, political considerations often overshadowed the supply chain considerations.

#### Lack of Data Quality, Availability, and Information Management System

The VCSP team observed a common issue across all countries, particularly in the initial months of the Initiative, which was lack of access to data, poor data completeness and quality, and/or delayed reporting. Specifically, unavoidable and avoidable vaccine wastages were least likely to be reported across countries. Although there has been some improvement in the quality and timeliness of reporting through structured data review, immunization programs still face systematic and consistent challenges with access to complete, quality, timely, and disaggregated data. Moreover, immunization programs in VCSP countries expressed concerns about the VCSP tool being a separate system instead of being integrated into existing logistics management information systems or health management information systems.

The VCSP team observed a common issue across all countries: lack of access to data, poor data completeness and quality, and delayed reporting.

## RECOMMENDATIONS

As the urgency of COVID-19 has waned and the epidemic has become endemic, immunization programs are shifting attention to integrating the COVID-19 vaccine into primary health care and routine immunization services, with this process still in its early phase of being rolled out. The lessons learned and benefits that were achieved through the VCSP Initiative provide a foundation for strengthening supply planning for all vaccines. Based on the insight from the VCSP Initiative related to the evolution of data systems, global and country-level experiences gained from COVID-19 vaccine supply chain management, and the readiness and interest of immunization programs, we offer the following recommendations for applying these approaches for improved supply planning and forecasting for all routine and new vaccines. Moving forward with both country and global recommendations is integral to the success of this approach.

### For Country Immunization Programs

- Incorporate and triangulate supply chain data with service delivery data for immunization supply chain decision-making. This reinforces the need for robust supply chain data systems, as noted by global thought leaders.^10^ Partners noted that the increased use of supply chain data from logistics management information systems and its triangulation with health management information systems data supported strengthened logistics management information systems in immunization, both for validating the data and its use in decision-making (e.g., ensuring that there were sufficient supplies to meet vaccination plans). Immunization programs acknowledged the benefit of the VCSP approach for the COVID-19 vaccine and expressed interest in applying similar approaches to routine vaccines and related supplies, and at the subnational level, in recognizing that the process would require long-term change management to adapt to routine vaccines. The VCSP Initiative approach was foundational for strengthening supply forecasting and planning for all vaccines, including for the integration of the COVID-19 vaccines into routine services.
- Evaluate multiple forecasting methods to improve the validity and accuracy of the final forecast. The incorporation of alternative forecasting methods using consumption or bottom-up analysis for routine vaccines, in addition to those using demographics and coverage goals, could result in more valid and accurate forecasts for all vaccines.^19^ This would be especially important for donors procuring vaccines and governments that self-finance or cofinance routine vaccines and enable them to evaluate more than 1 future scenario, resulting in increased accuracy, agility, and autonomy.
- Integrate the supply planning indicators and visuals demonstrated by the VCSP Initiative into the centralized system for supply chain management of all vaccines rather than as a stand-alone tool.
- Leverage existing relationships and the established trust of the collaborative model to continue the accelerated strengthening of supply chain management systems for vaccines. Partners who worked closely with the EPI and the NLWG, both within countries and across countries, noted that the collaborative nature of the VCSP Initiative was among its strengths.
- Enforce inventory management policies (e.g., minimum and maximum stock levels) for vaccine supply chain management and ensure adherence at all supply chain levels. As immunization programs mature, it may be timely to apply this standard in a practical way, learning from the COVID-19 experience to adopt into primary health care and routine immunization services.

### For Global Immunization Stakeholders

- Establish a global coordinating body to provide an unbiased forum for vested partners to review, predict, and identify potential discordance between vaccine supply and demand, as demonstrated by GFPVAN and the VCSP Initiative. A coordination body is instrumental in strengthening the preparedness of global supply chains to respond to our ever-changing global health environment (e.g., local manufacturing of vaccines, new vaccine introduction, shifting disease patterns, and future pandemics).^11^
- Establish a mechanism to gather standardized and real-time supply chain data for vaccine demand and supply across multiple countries. This mechanism would enable increased visibility, which is essential to effective and efficient global vaccine management (e.g., global forecasting, manufacturing, new product introduction, and equitable and appropriate distribution).^7^
- Adapt and expand global guidance to LMICs to incorporate contextualized local needs and the ever-evolving science of supply chain management to support more agile and responsive vaccine supply chains.

## CONCLUSIONS

The VCSP Initiative successfully applied best practices for supply planning, established a collaborative model with government decision-makers and partners, and used an adaptive learning approach to respond to the challenges of rolling out the COVID-19 vaccine. The Initiative increased visibility into country COVID-19 vaccine supply chain data, forecasting, and supply planning, leading to evidence-based decisions by countries on vaccine shipments and demand plans. Government stakeholders provided positive feedback on the Initiative’s success, feasibility, and acceptability and expressed interest in extending the approach to routine vaccines and to lower levels of their supply chain. Improving supply planning for all vaccines can further strengthen the overall immunization program as it continues to mature and provide a strong foundation for the integration of COVID-19 vaccines into primary health care and routine immunization services. The next steps will shift the focus to incorporating this approach to routine vaccines in a few follow-on countries to show its value, engaging global partners such as Gavi, UNICEF, and the World Health Organization to endorse potential changes. The VCSP Initiative’s success demonstrates the potential for a data-driven approach to supply planning to support immunization programs in achieving their goals.

## Supplementary Material

GHSP-D-23-00150-supplement2.pdf

GHSP-D-23-00150-supplement1.pdf

GHSP-23-00150-Akhlaghi-article-summary_French.pdf

GHSP-D-23-00150-supplement3.pdf

GHSP-23-00150-Akhlaghi-article-summary_Portuguese.pdf

GHSP-23-00150-Akhlaghi-article-summary_English.pdf

## Notes

**Peer Reviewed**

**First published online:** November 13, 2023.
