# Supplementary material for: COVID-19 Vaccine Collaborative Supply Planning: Is This the Next Frontier for Routine Immunization Supply Chains?
Source: Glob Health Sci Pract. 2024 Feb 20;12(Suppl 1):e2300150. doi: 10.9745/GHSP-D-23-00150 (PMC10948121; doi:10.9745/GHSP-D-23-00150)
Supplement: GHSP-D-23-00150-supplement2.pdf [file GHSP-D-23-00150-supplement2.pdf]

Leçons tirées de l'intégration de la vaccination contre la COVID-19 dans les soins de santé primaires | Série de résumés d'articles

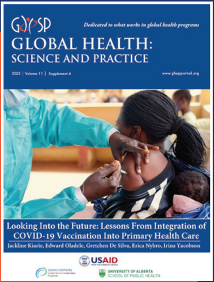

Planification concertée de l'approvisionnement en vaccins contre la COVID-19 : S'agit-il de la prochaine étape pour les chaînes d'approvisionnement en vaccins de routine ? Résumé de l'article

Laila Akhlaghi, Wendy Prosser, Avotiana Rakotomanga, Janet Makena, Tochukwu Azubike, Yahaya Bello, Samson Emelike, Liteboho Mothetsi, Moroke Motuba, Victor Olayemi, Sam Samba, Silvestre Suh, Stevens Ramaroson and Fatimata dit Ngo Yarro

De quoi parle cet article ? Les plans nationaux de déploiement du vaccin contre la COVID-19 dans les pays à revenu faible ou intermédiaire ont été élaborés en tenant compte de nombreuses incertitudes concernant l'offre, la demande, la disponibilité et les délais de fabrication. L'initiative de planification concertée des approvisionnements en vaccins (VCSP) a été créée pour contribuer à la coordination et à la planification de l'approvisionnement en vaccins contre la COVID-19 afin de s'assurer que les pays disposent des informations nécessaires pour prendre des décisions sur le moment et la quantité de vaccins disponibles.

Quels étaient les résultats ? L'outil de décision du VCSP a permis d'utiliser de manière innovante les données relatives à la prestation de services et à la chaîne d'approvisionnement, ce qui a aidé les gouvernements et les partenaires de mise en œuvre à vérifier les stocks de vaccins et à commander de nouvelles quantités si nécessaire, à suivre la consommation de vaccins, les déchets et les risques de péremption, ainsi qu'à planifier et coordonner les commandes de vaccins à venir. Le VCSP a créé un modèle de collaboration avec les

Points de vue des auteurs

John Snow, Inc. (JSI) a été le principal partenaire travaillant avec les ministères de la santé et les organisations de mise en œuvre pour développer, déployer et utiliser l'outil de décision VCSP. Le suivi, l'apprentissage et l'évaluation ont été assurés par inSupply Health Kenya. L'initiative VCSP a été financée par la Fondation Bill & Melinda Gates.

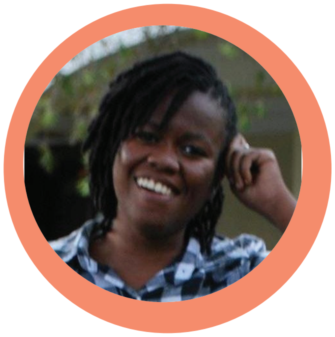

« Les partenaires ont noté que l'initiative VCSP a introduit des méthodes novatrices de gestion et d'analyse des données, ce qui s'est traduit par des avantages démontrables et a plaidé en faveur de l'intégration de ces méthodes novatrices de prévision et de planification des approvisionnements dans les efforts de vaccination de routine. »

-Janet Makena, conseillère MLE, inSupply Health Kenya

Lisez gratuitement l'article original publié dans Global Health Science and Practice à l'adresse suivante : https://doi.org/10.9745/GHSP-D-23-00150

décideurs gouvernementaux et les partenaires qui ont utilisé une approche d'apprentissage adaptatif pour apprendre en permanence et répondre aux défis du déploiement du vaccin contre la COVID-19.

Que signifient ces résultats ? Les résultats montrent comment les responsables des programmes de vaccination dans les pays peuvent utiliser cette approche de planification de l'approvisionnement pour planifier les vaccins nouveaux et de routine et renforcer la gestion de la chaîne d'approvisionnement en vaccins. Au niveau mondial, l'expérience du VCSP suggère que ce type d'initiative nécessite un soutien financier, politique et technique à plus long terme, un renforcement des capacités et une orientation globale.

Pourquoi l'initiative VCSP a-t-elle été créée ? La pandémie de COVID-19 a entraîné la mise au point rapide de vaccins, mais la capacité de production limitée et les perturbations des chaînes d'approvisionnement mondiales ont conduit à une fourniture et à une distribution inégale du vaccin dans le monde. Pour soutenir le déploiement du vaccin contre la COVID-19, l'initiative VCSP a été mise en place afin d'améliorer la situation :

- Prévision, processus d'estimation des quantités de vaccins contre la COVID-19 qui seront distribuées ou utilisées.
- La planification de l'approvisionnement, qui consiste à déterminer les quantités totales de produits nécessaires et à quel moment, sur la base des prévisions estimées, des niveaux de stock et des tendances et plans de consommation.

Quand et où l'initiative VCSP a-t-elle été mise en œuvre ? L'initiative VCSP a démarré en 2021, passant de 5 à 15 pays : République démocratique du Congo, Côte d'Ivoire, Éthiopie, Kenya, Lesotho, Madagascar, Malawi, Mali, Mozambique, Niger, Nigeria, Sierra Leone, Afrique du Sud, Tanzanie, et Ouganda.

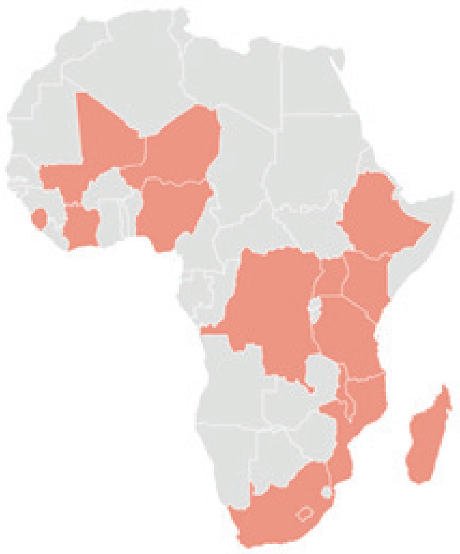

Quels sont les avantages de l'outil ?

- Les gestionnaires de la chaîne d'approvisionnement peuvent déterminer les quantités de fournitures et de commandes, vérifier l'état de l'approvisionnement et contrôler les performances.
- Les données relatives à la prestation de services pourraient être combinées avec les données relatives à la chaîne d'approvisionnement dans des visualisations afin d'aider les gestionnaires de programmes à prendre des décisions.
- Les pays disposaient d'informations sur les produits arrivant à expiration et pouvaient donner la priorité à la distribution de vaccins ou réaffecter des vaccins à d'autres pays.
- Les programmes pourraient suivre les schémas de consommation mensuelle des vaccins afin de mieux comprendre la demande future de vaccins et d'en optimiser la distribution.
- Les utilisateurs de l'outil peuvent créer des scénarios de planification de l'offre afin de réduire les risques potentiels liés à la chaîne d'approvisionnement.
- Cet outil a obligé les responsables gouvernementaux et les gestionnaires des programmes de vaccination à collaborer avec d'autres parties prenantes, ce qui a permis d'améliorer la coordination et d'accroître la confiance.
- Les équipes nationales pourraient mener une réflexion critique et analyser leur processus de planification des approvisionnements afin d'identifier les domaines à améliorer.

Quels ont été les défis à relever ?

- Les décisions politiques concernant les vaccins n'ont pas toujours été éclairées par les connaissances techniques tirées des données.
- Le manque d'accès aux données, la mauvaise qualité des données et l'absence de données précises sur le gaspillage des vaccins, bien que la qualité et l'actualité des données se soient améliorées au fil du temps.

USAID FROM THE AMERICAN PEOPLE

Knowledge SUCCESS

Ce guide a été réalisé grâce au soutien du peuple américain par l'intermédiaire de l'Agence américaine pour le développement international dans le cadre du projet Knowledge SUCCESS (Strengthening Use, Capacity, Collaboration, Exchange, Synthesis, and Sharing, accord de coopération n° 7200AA19CA00001 avec Johns Hopkins University. Knowledge SUCCESS est soutenu par le Bureau de la santé mondiale de l'USAID, le Bureau de la population et de la santé reproductive, et dirigé par le John Hopkins Centre for Communications Programs (CCP) en partenariat avec Amref Health Africa, le Centre d'économie comportementale de Busara et « FHI 360 ». Les informations fournies dans cette ressource relèvent de la seule responsabilité de Knowledge SUCCESS et ne reflètent pas nécessairement les opinions de l'USAID, du gouvernement américain ou de Johns Hopkins University.
