# Supplementary material for: COVID-19 Vaccine Collaborative Supply Planning: Is This the Next Frontier for Routine Immunization Supply Chains?
Source: Glob Health Sci Pract. 2024 Feb 20;12(Suppl 1):e2300150. doi: 10.9745/GHSP-D-23-00150 (PMC10948121; doi:10.9745/GHSP-D-23-00150)
Supplement: GHSP-D-23-00150-supplement1.pdf [file GHSP-D-23-00150-supplement1.pdf]

Lessons from Integration of COVID-19 Vaccination Into Primary Health Care | Article Summary Series

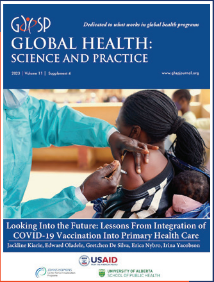

COVID-19 Vaccine Collaborative Supply Planning: Is This the Next Frontier for Routine Immunization Supply Chains? Article Summary

Laila Akhlaghi, Wendy Prosser, Avotiana Rakotomanga, Janet Makena, Tochukwu Azubike, Yahaya Bello, Samson Emelike, Liteboho Mothetsi, Moroke Motuba, Victor Olayemi, Sam Samba, Silvestre Suh, Stevens Ramaroson and Fatimata dit Ngo Yarro

What is this article about? National COVID-19 vaccine deployment plans in low- and middle-income countries were being developed with many unknowns about supply, demand, availability, and manufacturing timelines. The Vaccine Collaborative Supply Planning (VCSP) Initiative was created to help with the coordination and planning of the COVID-19 supply to make sure countries had information to make decisions based on vaccine availability.

What were the findings? The VCSP decision tool provided several innovative ways of using service delivery and supply chain data that helped governments and implementing partners check vaccine stock and reorder quantities as necessary, track vaccine consumption, waste, and potenial for expiry, and coordinate future vaccine orders. The VCSP created a collaborative model with government decision-makers and partners that used an adaptive learning approach to continuously learn and respond to the challenges of rolling out the COVID-19 vaccine.

What do these results mean? The findings show how country-based immunization program managers can use this supply planning approach to plan for new and routine vaccines and strengthen the management of the immunization supply chain. At the global level, the VCSP experiences suggests that this type of initiative requires longer-term financial, policy, and technical support, capacity-strengthening, and global guidance.

Insights from the Authors

John Snow, Inc. (JSI) was the lead partner working with MOHs and implementing organizations to develop develop, deploy, and use the VCSP decision tool. Monitoring, Learning and Evaluation support was provided by inSupply Health Kenya. The VCSP Initiative was funded by the Bill & Melinda Gates Foundation.

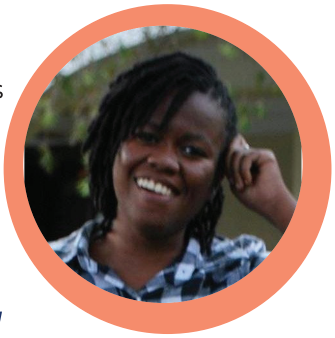

"Partners noted that the VCSP Initiative introduced innovative ways of data management and analysis, resulting in demonstrable benefits and making the case for integrating these innovative methods of forecasting and supply planning into routine immunization efforts."

-Janet Makena, MLE Advisor, inSupply Health Kenya

Read the original article published in Global Health Science and Practice for free at:
https://doi.org/10.9745/GHSP-D-23-00150

Why was the VCSP initiative created? The COVID-19 pandemic prompted the rapid development of vaccines, but limited manufacturing capacity and disruption in global supply chains resulted in uneven supply and distribution of the vaccine globally. To support the roll-out of the COVID-19 vaccine, the VCSP Initiative was established to address to improve:

- Forecasting , the process of estimating the quantities of COVID-19 vaccines that would be dispensed or used.
- Supply planning, determining the total quantities of products needed and when, based on the forecasting estimate, stock levels, and consumption plans and trends.

When and where was the initiative implemented? The VCSP Initiative started in 2021, expanding from 5 to 15 countries: Democratic Republic of Congo, Cote d'Ivoire, Ethiopia, Kenya, Lesotho, Madagascar, Malawi, Mali, Mozambique, Niger, Nigeria, Sierra Leone, South Africa, Tanzania, Uganda.

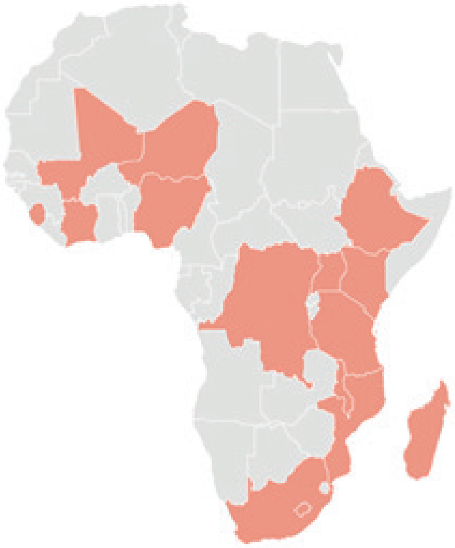

What were some benefits of the tool?

- Supply chain managers could determine quantities of supplies and orders, check supply status, and monitor performance.
- Service delivery data could be combined with supply chain data in visualizations to help program managers with decision-making.
- Countries had insights on products that were going to expire and could prioritize vaccines for distribution or reallocate vaccines to other countries.
- Programs could track monthly vaccine consumption patterns to help understand future demand for the vaccine and optimize distribution.
- Users could create supply planning scenarios to help reduce potential supply chain risks.
- The tool required government leaders and immunization program managers to work together with other stakeholders, which improved coordination and increased trust.
- Country teams could critically reflect and analyze their supply planning process to identify areas of improvement.

What were some challenges?

- Political decisions about vaccines were not always informed by the technical insight gained from the data.
- Lack of access to data, poor-quality data, and and lack of accurate data on vaccine wastage, though the quality and timeliness of data improved as time went on.

USAID FROM THE AMERICAN PEOPLE

Knowledge SUCCESS

This summary brief is made possible by the support of the American People through the U.S. Agency for International Development under the Knowledge SUCCESS (Strengthening Use, Capacity, Collaboration, Exchange, Synthesis, and Sharing) Project Cooperative Agreement No. 7200AA19CA00001 with the Johns Hopkins University. Knowledge SUCCESS is supported by USAID's Bureau for Global Health, Office of Population and Reproductive Health and led by the Johns Hopkins Center for Communication Programs (CCP) in partnership with Amref Health Africa, The Busara Center for Behavioral Economics (Busara), and FHI 360. The information provided in this summary brief are the sole responsibility of Knowledge SUCCESS and does not necessarily reflect the views of USAID, the U.S. Government, or the Johns Hopkins University.
