# Supplementary material for: COVID-19 Vaccine Collaborative Supply Planning: Is This the Next Frontier for Routine Immunization Supply Chains?
Source: Glob Health Sci Pract. 2024 Feb 20;12(Suppl 1):e2300150. doi: 10.9745/GHSP-D-23-00150 (PMC10948121; doi:10.9745/GHSP-D-23-00150)
Supplement: GHSP-D-23-00150-supplement3.pdf [file GHSP-D-23-00150-supplement3.pdf]

Lições da integração da vacinação contra a COVID-19 nos cuidados de saúde primários | Série de resumos de artigos

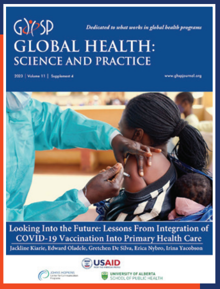

Planeamento colaborativo do fornecimento de vacinas contra a COVID-19: será esta a próxima fronteira para as cadeias de abastecimento de imunização de rotina? Resumo do artigo

Laila Akhlaghi, Wendy Prosser, Avotiana Rakotomanga, Janet Makena, Tochukwu Azubike, Yahaya Bello, Samson Emelike, Liteboho Mothetsi, Moroke Motuba, Victor Olayemi, Sam Samba, Silvestre Suh, Stevens Ramaroson and Fatimata dit Ngo Yarro

De que trata este artigo? Os planos nacionais de distribuição da vacina contra a COVID-19 nos países de baixo e médio rendimento estavam a ser desenvolvidos com muitas incógnitas sobre a oferta, a procura, a disponibilidade e os prazos de fabrico. A Iniciativa de Planeamento Colaborativo do Abastecimento de Vacinas (VCSP) foi criada para ajudar na coordenação e planeamento do abastecimento de vacinas contra a COVID-19, a fim de garantir que os países dispunham de informações que lhes permitissem tomar decisões sobre quando e quantas vacinas estavam disponíveis.

Quais foram as conclusões? A ferramenta de decisão VCSP forneceu várias formas inovadoras de utilização de dados sobre a prestação de serviços e a cadeia de abastecimento que ajudaram os governos e os parceiros de implementação a verificar o stock de vacinas e a voltar a encomendar as quantidades necessárias, a acompanhar o consumo de vacinas, o desperdício e o potencial de expiração, e a planear e coordenar futuras encomendas de vacinas. O VCSP criou um modelo de colaboração com decisores e parceiros governamentais que utilizou uma abordagem de aprendizagem adaptativa

Perspetivas dos autores Points de vue des auteurs

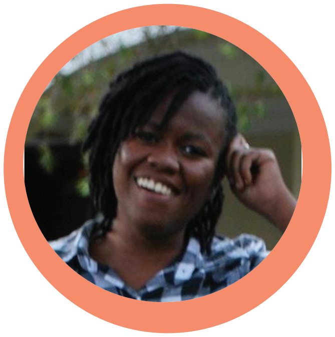

A John Snow, Inc. (JSI) foi o parceiro principal que trabalhou com os Ministérios da Saúde e as organizações de implementação para desenvolver, implementar e utilizar a ferramenta de decisão VCSP. O apoio à monitorização, aprendizagem e avaliação foi prestado pela inSupply Health Kenya. A iniciativa VCSP foi financiada pela Fundação Bill & Melinda Gates.

> ***"Os parceiros observaram que a Iniciativa VCSP introduziu formas inovadoras de gestão e análise de dados, resultando em benefícios demonstráveis e defendendo a integração destes métodos inovadores de previsão e planeamento de fornecimento nos esforços de imunização de rotina."***
>
> ***-Janet Makena***, Conselheira MLE, inSupply Health Kenya

Leia o artigo original publicado na Global Health Science and Practice gratuitamente em: https://doi.org/10.9745/GHSP-D-23-00150

para aprender continuamente e responder aos desafios da implementação da vacina contra a COVID-19.

O que significam estas conclusões? Os resultados mostram como os gestores de programas de imunização baseados nos países podem utilizar esta abordagem de planeamento do abastecimento para planear vacinas novas e de rotina e reforçar a gestão da cadeia de abastecimento de imunização. A nível mundial, a experiência do VCSP sugere que este tipo de iniciativa requer apoio financeiro, político e técnico a longo prazo, reforço das capacidades e orientação global.

Por que razão foi criada a Iniciativa VCSP? A pandemia de COVID-19 levou ao rápido desenvolvimento de vacinas, mas a capacidade de fabrico limitada e as perturbações nas cadeias de abastecimento mundiais resultaram num fornecimento e distribuição desiguais da vacina a nível mundial. Para apoiar o lançamento da vacina contra a COVID-19, foi criada a Iniciativa VCSP, com o objetivo de melhorar:

- A previsão, o processo de estimar as quantidades de vacinas contra a COVID-19 que seriam distribuídas ou utilizadas.
- O planeamento do abastecimento, determinando as quantidades totais de produtos necessários e quando, com base na estimativa de previsão, nos níveis de stock e nas tendências e planos de consumo.

Quando e onde foi implementada a Iniciativa VCSP? A iniciativa VCSP teve início em 2021, expandindo-se de 5 para 15 países: República Democrática do Congo, Costa do Marfim, Etiópia, Quénia, Lesoto, Madagáscar, Malawi, Mali, Moçambique, Níger, Nigéria, Serra Leoa, África do Sul, Tanzânia e Uganda.

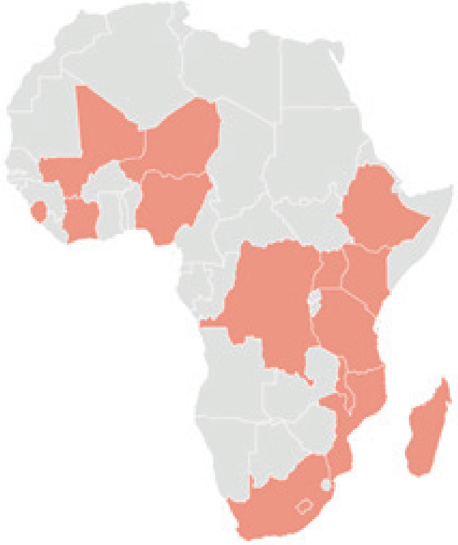

Quais foram os benefícios da ferramenta?

- Os gestores da cadeia de abastecimento podiam determinar as quantidades de fornecimentos e encomendas, verificar o estado dos fornecimentos e monitorizar o desempenho.
- Os dados de prestação de serviços podiam ser combinados com os dados da cadeia de abastecimento em visualizações para ajudar os gestores de programas na tomada de decisões.

- Os países tinham informações sobre os produtos que iam expirar e podiam dar prioridade às vacinas para distribuição ou reafetar vacinas a outros países.
- Os programas podiam acompanhar os padrões mensais de consumo de vacinas para ajudar a compreender a futura procura da vacina e otimizar a distribuição.
- Os utilizadores da ferramenta podiam criar cenários de planeamento do abastecimento para ajudar a reduzir os potenciais riscos da cadeia de abastecimento.
- A ferramenta exigiu que os líderes governamentais e os gestores de programas de imunização trabalhassem em conjunto com outras partes interessadas, o que melhorou a coordenação e aumentou a confiança.
- As equipas nacionais podem refletir criticamente e analisar o seu processo de planeamento do aprovisionamento para identificar áreas de melhoria.

Quais foram os desafios?

- As decisões políticas sobre as vacinas nem sempre foram informadas por conhecimentos técnicos obtidos a partir dos dados.
- Falta de acesso aos dados, dados de má qualidade e falta de dados exatos sobre o desperdício de vacinas, embora a qualidade e a atualidade dos dados tenham melhorado com o passar do tempo.

Este guia resumido é possível graças ao apoio do povo americano através da U.S. Agency for International Development ao abrigo do Acordo de Cooperação do Projeto Knowledge SUCCESS (Strengthening Use, Capacity, Collaboration, Exchange, Synthesis, and Sharing) n.º 7200AA19CA00001 com a Universidade Johns Hopkins. O Knowledge SUCCESS é apoiado pelo Gabinete de Saúde Global, da População e Saúde Reprodutiva da USAID e dirigido pelo Johns Hopkins Center for Communication Programs (CCP) em parceria com a Amref Health Africa, The Busara Center for Behavioral Economics (Busara) e a FHI 360. As informações fornecidas neste guia resumido são da exclusiva responsabilidade da Knowledge SUCCESS e não refletem necessariamente as opiniões da USAID, do Governo dos EUA ou da Universidade Johns Hopkins.
